# Supplementary material for: Non-dominant hand contractions do not facilitate performance under pressure in common desktop tasks
Source: PLoS One. 2025 Jan 22;20(1):e0316355. doi: 10.1371/journal.pone.0316355 (PMC11753714; doi:10.1371/journal.pone.0316355)
Supplement: Attachment — Submitted filename: response_letter.docx [file pone.0316355.s001.docx]

We thank the reviewer for considering our manuscript and their comments.

we recommend adding relevant (numerical) results, not just the textual interpretation.

***This has been addressed***

Introduction:
- The novel aspects of the present study should be detailed more concretely in relation to previous studies on the same topic.

***Our three studies test the after-affects of non-dominant hand contractions in common desktop motor tasks performed under pressure. To our knowledge, this has not been attempted before. Previous work has considered the context of elite music or sport.***

***We have edited the text starting line 151 to more concretely state this:***

***To date, researchers have considered the after-effects of NDHCs benefits in elite sport and music, with little consideration of the after-effects in non-elite domains. Accordingly, we tested the benefits of NDHCs on well-known (“everyday”) computer tasks conducted under time and/or performance pressure.***

The presentation of the two studies is detailed, but I think it could be compressed, the manuscript has 45 pages, which is much too long for an article.

**We shortened the manuscript throughout and have removed ~750 words. Please note that we have presented three studies rather than two.**

- to add the practical implications based on the results of the study.

**All studies found null results which limits the implications we can offer. It would be possible to suggest that NDHCs should not be used in desktop tasks performed under pressure, but this is not very practical. Had we found significant results we could have offered practical implications. Our results question a common finding in sport-based studies and suggest that the NDHC phenomenon requires more explanation and research (next point).**

- to compare the conclusions with 2-3 more relevant ideas based on the relevant results of the study and possibly to identify future research directions.

***We have suggested future research in a distributed fashion throughout the discussion on lines 658, 662, 669 and 681.***
